# Developing and refining the COVID-19 to Routine Immunization Information System Transferability Assessment (CRIISTA) tool: a decision support tool to leverage COVID-19 immunization information system investments for routine immunization

**DOI:** 10.1093/oodh/oqae006

**Published:** 2024-05-06

**Authors:** Nami Kawakyu, Allison Osterman, Jimi Michel, Dominic Mutai, Edith Jepleting, Grace Njenga, Parysa Oskouipour, Jessica C Shearer

**Affiliations:** MOMENTUM Routine Immunization Transformation and Equity, PATH, 2201 Westlake Avenue, Seattle, WA 98121, USA; MOMENTUM Routine Immunization Transformation and Equity, PATH, 2201 Westlake Avenue, Seattle, WA 98121, USA; MOMENTUM Routine Immunization Transformation and Equity, John Snow Inc, 44 Farnsworth St, Boston, MA 02210, USA; Living Labs, PATH, ACS Plaza, 4th floor. Lenana Road, Nairobi, Kenya; Living Labs, PATH, ACS Plaza, 4th floor. Lenana Road, Nairobi, Kenya; Living Labs, PATH, ACS Plaza, 4th floor. Lenana Road, Nairobi, Kenya; MOMENTUM Routine Immunization Transformation and Equity, John Snow Inc, 44 Farnsworth St, Boston, MA 02210, USA; MOMENTUM Routine Immunization Transformation and Equity, PATH, Washington, DC, USA

**Keywords:** COVID-19, routine immunization, health information system, digital health, decision support tool, scale-up

## Abstract

To achieve the Immunization Agenda 2030 vaccine equity vision of a world where everyone everywhere benefits from vaccines, it is essential to invest in immunization information systems that can support the identification, reach and monitoring of zero-dose and under-immunized children. The rapid nature of COVID-19 vaccine introduction led to the investment of new systems to collect, manage and use immunization data. While many digital health assessment tools exist, there is an absence of tools to support decision-makers to systematically assess the suitability of transferring an immunization information system from one health context to another. To address this gap, the COVID-19 to Routine Immunization Information System Transferability Assessment tool was developed, informed by literature review, expert consultation and usability testing. The tool is organized into five thematic areas: context, functionality, technology, users and resources. Each thematic area has questions about the COVID-19 immunization information system, the current and desired state of the routine immunization information system and the gaps between them. Suitability scores are then calculated to assess whether a COVID-19 immunization information system is suitable for routine immunization so governments can leverage these investments to strengthen routine immunization programs and the broader health information ecosystem.

Abrégé

Pour réaliser la vision du Programme pour la vaccination à l’horizon 2030 concernant l’équité vaccinale d’un monde où tout le monde, partout, bénéficie des vaccins, il est essentiel d’investir dans des systèmes d’information sur la vaccination capables d’appuyer l’identification, l’accès et le suivi des enfants zéro dose et insuffisamment vaccinés. La nature rapide de l’introduction du vaccin contre la COVID-19 a entraîné l’investissement dans de nouveaux systèmes de collecte, de gestion et d’utilisation des données sur la vaccination. Bien que de nombreux outils numériques d’évaluation de la santé existent, on ne dispose d’aucun outil pour aider les décideurs à évaluer systématiquement la pertinence du transfert d’un système d’information sur la vaccination d’un contexte de santé à un autre. Pour combler cette lacune, l’outil d’évaluation de la transférabilité de la COVID-19 au système d’information sur la vaccination de routine a été élaboré, éclairé par un examen documentaire, des consultations d’experts et des tests bêta. Cet outil s’articule autour de cinq domaines thématiques: contexte, fonctionnalité, technologie, utilisateurs et ressources. Chaque domaine thématique comprend des questions sur le système d’information sur la vaccination contre la COVID-19, l’état actuel et souhaité du système d’information sur la vaccination de routine et les écarts entre eux. Les scores de pertinence sont ensuite calculés pour évaluer si un système d’information sur la vaccination contre la COVID-19 convient à la vaccination de routine afin que les pouvoirs publics puissent tirer parti de ces investissements pour renforcer les programmes de vaccination de routine et l’écosystème de l’information sur la santé dans son ensemble.

Resumen

Para lograr la visión de la Agenda de Inmunización 2030 de un mundo donde todas las personas de todas partes se beneficien de las vacunas, es esencial invertir en sistemas de información sobre vacunación que puedan apoyar la identificación, el alcance y el monitoreo de niños con dosis cero y subvacunados. La rápida introducción de la vacuna COVID-19 condujo a la inversión en nuevos sistemas para recopilar, administrar y utilizar datos sobre vacunación. Si bien existen muchas herramientas digitales de evaluación de la salud, faltan instrumentos para ayudar a los responsables de la toma de decisiones a evaluar sistemáticamente la idoneidad de transferir un sistema de información sobre vacunación de un contexto sanitario a otro. Para corregir esta deficiencia, se creó el Marco de evaluación de la transferibilidad del sistema de información sobre la vacunación contra la COVID-19 a la vacunación sistemática (CRIISTA), sobre la base de la revisión de literatura, consultas a expertos y pruebas beta. El Marco está organizado en cinco áreas temáticas: contexto, funcionalidad, tecnología, usuarios y recursos. Cada área temática contiene preguntas referidas al sistema de información sobre la vacunación contra la COVID-19, el estado actual del sistema de información sobre la vacunación sistemática y su estado deseado, y las brechas entre ellos. Seguidamente, se calculan las puntuaciones de idoneidad para evaluar si un sistema de información sobre la vacunación contra la COVID-19 es adecuado para la vacunación sistemática, de modo que los Gobiernos puedan aprovechar estas inversiones para fortalecer los programas de vacunación sistemática y el sistema más amplio de información sanitaria.

## INTRODUCTION

Despite considerable progress in reducing global child mortality, gains have not been equal across or within countries, and challenges remain [1]. These challenges include stalled progress in expanding routine immunization (RI) services to reach all children [2], with many countries’ childhood immunization coverage below the ≥90% goal set by the World Health Organization (WHO) [3–5]. This was exacerbated by the COVID-19 pandemic, which contributed to the largest sustained decline in global childhood vaccination coverage in three decades [6]. In 2021 alone, nearly 18 million infants worldwide did not receive the initial dose of diphtheria tetanus pertussis (DTP) vaccine and another 7 million were only partially vaccinated [3]. These ‘zero-dose children’, i.e. children who have not received any routine vaccine [2], and under-immunized children are left vulnerable to infectious diseases that can cause serious illness, disability and death.

Reaching zero-dose and under-immunized children, who mostly live in urban areas, remote communities or conflict settings [2, 4], requires information about who is missed and why. As one part of a holistic data use ecosystem, immunization information systems (IISs) need to support its users to be able to identify where these children live, in what numbers, and for what reasons they are zero-dose or under-immunized [7, 8]. Investing in IISs to capture, analyse and use this data will enable health professionals and other stakeholders to improve RI services to achieve the Immunization Agenda 2030 vaccine equity vision of ‘a world where everyone, everywhere, at every age, fully benefits from vaccines to improve health and well-being’ [9].

In many countries, efforts to introduce and scale up IISs for RI have faced constraints related to the country’s digital health enabling environment [10]. Challenges related to funding, infrastructure and leadership and governance hindered the widespread adoption of IISs such as electronic immunization registries [11–13]. The rapid development and introduction of the COVID-19 vaccine led to massive investments in new systems, infrastructure and human resources to collect, manage and use COVID-19 immunization data [14–16]. National governments and their partners, having invested heavily in COVID-19 IISs, are grappling with the desire to leverage these investments for the RI context while concerned that this translation may not be appropriate, feasible or cost-effective. For example, the political will, human and financial resources and hardware made available to support the adoption and use of IISs for COVID-19 vaccination may not be available for RI. The functional requirements of a COVID-19 and RI IIS differ in terms of client age groups, clinical workflows and clinical decisions to be made. These are two examples of how even a successful COVID-19 IIS may not be easily transferred for RI and reflect the types of questions posed by national immunization and digital health decision-makers to the United States Agency for International Development (USAID)-funded MOMENTUM Routine Immunization Transformation and Equity project (‘the project’) in 2022 and 2023.

This paper is part of a larger collaborative learning agenda documenting whether and how digital investments made as part of the COVID-19 vaccination emergency response contributed to strengthening health systems beyond their immediate intended outcomes [17, Figure 1], e.g. whether investments in COVID-19 IISs contributed to strengthening the broader IIS. The authors involved in this larger learning agenda developed a theory of change [17] that hypothesizes that emergency digital investments could lead toward more durable impact by strengthening the digital health enabling environment, and that successful institutionalization of emergency IISs may also require certain elements of the digital health enabling environment to be present at adequate levels. This reflects lessons learned from past deployments and scale-up of electronic health information systems (HISs), which indicate the importance of planning, with careful consideration of the political and digital health enabling environments [10, 18, 19]. Similar to the COVID-19 context, during the Ebola outbreak in 2013, there was a surge of funding for digital health solutions; however, post-Ebola, these interventions largely fell into disuse [20]. A key lesson learned from this experience was the need for better planning regarding how these surge investments can be leveraged for other health areas or sustained at the end of emergency pandemic funding [20]. Had decision support tools and processes for transferring and scaling up these investments to different contexts been available to decision-makers, these interventions may have been better integrated and sustained post-Ebola and contributed to a strengthened digital health enabling environment [20].

Given this, a systematic assessment of the potential suitability of these COVID-19 IISs for transfer and scale-up to the RI context is required, as well as an understanding of the conditions necessary for successful transfer and scale-up. While many assessment frameworks have been developed for digital health [21–23] and scale-up [24, 25], we did not find one that was adequately tailored for assessing a specific IIS. Most of the existing digital health frameworks broadly assess country readiness to introduce digital health solutions. None of the existing frameworks focus on taking an IIS that has been implemented in one context and transferring or scaling it up to another context or use case, noting the differences in needs and resources between the two.

To this end, the project developed the COVID-19 to Routine Immunization Information System Transferability Assessment (CRIISTA) tool to support decision-makers to systematically assess the suitability of transferring and scaling up a COVID-19 IIS for RI. This paper presents the process of developing, usability testing and revising the tool and describes the resulting final tool. The assessment tool described in this paper aimed to contribute to the realization of the learning agenda’s theory of change assumptions, and ultimately outcomes, by supporting conversations that directly address the sustainability of COVID-19 vaccine-related digital investments.

## METHODS

The CRIISTA tool was developed and refined through the project across three phases: tool development, tool usability testing and tool revision. We briefly describe the methods for each phase below, followed by the outcomes of each in the results section.

### Phase 1. Tool development

#### Literature review

We conducted a literature review to identify tools and frameworks for assessing digital health interventions and HISs that could be used to inform the development of the CRIISTA tool. The literature search was conducted from March to May 2022 using PubMed for peer-reviewed literature and Google for grey literature, including reports, assessment tools and frameworks. The keyword search included variations of the following search terms: digital health intervention, immunization information system, readiness, maturity, scale-up and terms related to components of the WHO and the International Telecommunication Union eHealth Strategy building blocks [10]. The study team also consulted immunization and digital health experts to identify relevant tools, frameworks and literature. Tools and frameworks were included in the review if they provided considerations for making decisions about introducing and/or scaling up a health intervention, including both digital and non-digital interventions.

The included tools and frameworks were reviewed by the tool development team composed of four senior researchers with expertise in immunization and HISs. A subset of tools and frameworks was selected to inform the development of the draft CRIISTA tool. Tools and frameworks were selected if they addressed any one of the following areas: (1) digital health enabling environment; (2) conditions for successful scale-up; (3) obstacles for implementation; (4) suitability of the intervention within the proposed context; and (5) value add or usefulness of the intervention for the new use case.

A thematic analysis of the selected tools and frameworks was conducted to identify commonalities and differences related to use case, key attributes and assessment categories. Descriptive information was extracted from each tool or framework and organized in a matrix for analysis.

#### Tool development

The tool development team drafted the initial CRIISTA tool and specified its target users and intended use cases. The team drew on relevant assessment themes and questions from the selected tools and frameworks. Key considerations and elements for assessing the suitability and feasibility of an intervention for scale-up were incorporated into the CRIISTA tool. Questions in the tool were further adapted by the team to relate specifically to digital health interventions for improving immunization data management. The tool development team discussed the layout and format of reviewed tools to inform the layout and user interface for the CRIISTA tool.

The draft tool was then refined between June and July 2022, following review by nine immunization, digital health and health systems experts in the Democratic Republic of the Congo (DRC), Kenya and the United States. Experts in the DRC and Kenya were invited to review the draft tool because the project works in both countries and, at the time, both countries’ ministries of health had expressed potential interest in scaling up or transferring their COVID-19 IIS for RI.

### Phase 2. Tool usability testing

To test the feasibility and usability of the tool (usability testing), the tool development team collaborated with Kenyan-based digital health and immunization data experts (the implementation team) from July to December 2022.

The aim of usability testing was to inform improvements to the tool and the tool implementation process. Specific usability testing objectives included:

1. Collect data required to complete the tool questions through document review and interviews with immunization, digital health and HIS experts.
2. Identify what worked well with the tool itself, including whether the questions were clear and whether they solicited the information that decision-makers need to make an informed decision about transferring their COVID-19 IIS for RI.
3. Document the tool implementation process, including how the responses to tool questions were obtained and how much time was required to collect and synthesize the data.

#### Setting

Kenya was selected because the Ministry of Health was considering transferring their COVID-19 electronic immunization registry, Chanjo Kenya System (Chanjo Kenya), for RI, and the tool development team anticipated there would be political will and interest to systematically assess the feasibility of a transfer.

Chanjo Kenya was jointly developed by Kenya’s Ministry of Health and Ministry of Information and Communication Technology and was deployed in 2021 as a digital COVID-19 client immunization registry to register and record individual client demographic and vaccination information, send SMS text reminders for subsequent doses and provide a digital vaccination certificate. Health workers and health system data managers entered data into Chanjo Kenya using a tablet in either web-based or offline modes. Chanjo Kenya was later introduced as a self-service online portal that allows users to register for vaccinations, choose their preferred vaccination centres and view vaccination schedules, as well as download and verify their COVID-19 vaccination certificates.

While a digital IIS was deployed for COVID-19, RI data in Kenya is recorded and managed using paper-based tools at the community and facility level. Nurses enter client demographic data and data on doses administered in an immunization register as they immunize children. These register books include a row for each child in the catchment area with spaces to enter each vaccine administered. This widely used system has well-documented challenges for health workers, which limit their ability to use data for clinical decision-making and for sending reminders—steps that could help to improve coverage and reduce dropout [26]. At the end of the month, the data on vaccines administered and children immunized is tallied. The nurse supervisors then transmit the monthly aggregate totals to the sub-county level where they are entered by a data entry clerk into the Kenya Health Information System, which is supported by DHIS2. The system provides aggregate and summary reports of the data, which can be accessed by various Ministry of Health staff at national, county and sub-county levels. The Ministry of Health attributes sub-optimal collection, analysis, use and overall management of immunization data as a key barrier to identifying how many children are eligible for immunization services and how many are under-vaccinated [27]. Without this accurate information, it is difficult to plan immunization services and provide them at the right places at the right times.

#### Data collection and analysis

##### Using CRIISTA (objective 1)

As recommended in the CRIISTA tool, the implementation team answered the questions in the tool through document review and semi-structured interviews. The implementation team first reviewed documents that described or evaluated the Chanjo Kenya deployment for COVID-19 immunization data management and extracted relevant data to answer the tool assessment questions. Next, the implementation team conducted interviews with purposively selected immunization, digital health and HIS experts from the Kenya Ministry of Health to answer questions that were not addressed by the document review. Key informants were convened during a 1-hour expert panel interview, held via videoconference in November 2022.

##### Evaluating CRIISTA’s usability and feasibility (objectives 2 and 3)

To assess tool usability and implementation feasibility, the tool development team reviewed documents outlining the process of using the tool, the CRIISTA tool filled with Kenya data and implementation teams’ notes on the experience of using the CRIISTA tool.

‘Pause and Reflect’ sessions, an adaptive management method [28–30], were also conducted to qualitatively assess tool usability and implementation feasibility. These sessions generated data on what was done, what worked well and what could be improved. The first pause and reflect session was held with the tool development team in November 2022. The second pause and reflect session was held in December 2022 with the tool development team and implementation team, guided by a semi-structured data collection tool. Pause and reflect data was analyzed for themes, guided by the usability testing aims.

**Table 1:** Tools and frameworks reviewed

	Tools and frameworks reviewed	Digital health framework	Non-digital health framework	Immunization-specific framework
1	Data Use Acceleration and Learning Implementation Framework [32]	x		
2	Digital Health Atlas [33]	x		
3	Digital Health Investment Review Tool [34]	x		
4	Digital Implementation Investment Guide [22]	x		
5	Early Stage Digital Health Investment Tool [23]	x		
6	Electronic Immunization Registry Readiness Assessment Tool [31]	x		x
7	Global Digital Health Monitor (formerly the Global Digital Health Index) [35]	x		
8	Health Information System Interoperability Maturity Toolkit [36]	x		
9	Health Information System Stages of Continuous Improvement Toolkit [37]	x		
10	Intervention Scalability Assessment Tool [24]		x	
11	mHealth Assessment and Planning for Scale Toolkit [21]	x		
12	National eHealth Strategy Building Blocks [10]	x		
13	Navigator for Digital Health Capability Models [38]	x		
14	One Health Information System Maturity Assessment Tool [39]	x		
15	Performance of Routine Information System Management Framework [40]	x		

### Phase 3. Tool revision and user guide development

Informed by findings from the Kenya usability testing, the tool development team took a systematic approach to revise the tool from December 2022 to January 2023. Revisions were made to the overall structure of the tool, mapping the questions from the initial version to the new structure, and revising open-ended questions to close-ended questions. The revised tool was further refined following feedback from immunization, digital health and HIS experts from the project and other technical partners across six countries in Africa, Asia and North America. Five experts provided in-depth feedback and over a dozen provided feedback and comments during presentations delivered by the tool development team, which informed tool revisions.

**Table 2:** Existing tools and frameworks used to inform the development of CRIISTA

	Digital health frameworks						Public health framework
	**Digital Health Investment Review Tool**	**Early Stage Digital Health Investment Tool**	**Electronic Immunization Registry Readiness Assessment Tool**	**Global Digital Health Monitor (formerly the Global Digital Health Index)**	**Health Information System Interoperability Maturity Toolkit**	**mHealth Assessment and Planning for Scale Toolkit**	**Intervention Scalability Assessment Tool**
Assessment areas addressed^a^	1–4	1–4	1–4	1–3	1–4	1–4	1–5
Use cases	Inform best practices for investments in digital technology	Assess digital health intervention readiness	Determine country’s current capacity for successful and sustainable electronic immunization record implementation	Benchmark and monitor countries’ effective use of digital health investments	Identify country’s status according to required levels of health information system maturity to achieve interoperability goals	Assess progress of scaling up an already deployed mHealth product and plan next steps for scale-up	Determine scalability of discrete health program or intervention; identify and assess contextual factors helping or hindering scale-up
Structure	12 elements that are scored on a 1–5 scale	Six essential building blocks with 19 sub-categories and 71 total indicators categorized as information, enabling, or critical	Six categories with 12 sub-categories and 62 total indicators categorized as informational, enabling, or critical	Seven key indicator categories	Three key domains and multiple sub-domains	Six axes of scale and 16 sub-domains	Two parts, each with 5 domains
Assessment categories	eHealth policy landscape; health information system ecosystem; key stakeholders; system users and relevant groups; scale; cost of ownership; monitoring, evaluation and learning plan; open vs proprietary; privacy and security; reuse and improve; change management	Human capacity; standards and interoperability; governance and policy; data capture and use; investments and funding; infrastructure	Human capacity; standards and interoperability; governance and policy; process and information sources; investments and funding; infrastructure	Leadership and governance; strategy and investment; legislation, policy and compliance; workforce; standards and interoperability; infrastructure services and applications	Leadership and governance; human resources; technology	Groundwork; partnerships; financial health; technology and architecture; operation; monitoring and evaluation assessment	Problem; intervention; context; evidence of effectiveness; intervention costs; fidelity and adaptation; reach and acceptability; delivery setting; infrastructure; sustainability

a Assessment areas: (1) digital health enabling environment, (2) conditions for successful scale-up, (3) obstacles for implementation, (4) suitability of intervention within proposed context and (5) value-add or usefulness of intervention for new use case.

## RESULTS

### Phase 1. Tool development

#### Literature review and expert consultation

The literature search yielded a total of 22 articles, including 15 tools and frameworks that were reviewed by the tool development team (Table 1). Of the 15 tools and frameworks, 14 (93%) relate to assessing digital health solutions and one (7%) relates to assessing the suitability of health interventions for scale-up. All but three (*n* = 12, 80%) assess the overall digital health enabling environment within a given country as opposed to a specific digital health intervention. Only two (13%), the mHealth Assessment and Planning for Scale Toolkit and the Intervention Scalability Assessment Tool, provide a framework for the scale-up process. Just one among the 14 digital health-related tools and frameworks, the Electronic Immunization Registry Readiness Assessment Tool, focuses on assessing country readiness to adopt or scale up an immunization digital health intervention. This tool provides a framework for countries to determine their current capacity to successfully and sustainably implement an IIS [31]. While it has many parallels to current decision-making related to readiness to transfer or scale up COVID-19 IISs, it and most other frameworks were developed prior to COVID-19 and do not account for the contextual factor central to this use case; that is, that an intervention is developed and implemented in an emergency context with higher levels of resources and political will.

Based on the analysis and identification of common themes across the gathered literature, we identified five broad assessment areas that were relevant to inform decision-making and were determined to be essential to be included in the tool: (1) digital health enabling environment, (2) conditions for successful scale-up, (3) obstacles for implementation, (4) suitability of the intervention within the proposed context and (5) value-add or usefulness of the intervention for the new use case.

Of the 15 tools and frameworks reviewed, a subset of seven (47%) was used to inform the development of the initial CRIISTA tool because it addressed one or more of the identified assessment areas. Table 2 indicates which assessment areas were addressed by each of the selected tools and frameworks and describes each tool’s or framework’s use cases, structure and assessment categories.

#### Draft CRIISTA tool

The tool development team drafted the initial version of the CRIISTA tool by using relevant assessment questions from seven frameworks and adapting them to relate to digital health interventions for improving RI data management.

The draft tool included 43 questions across four thematic areas: (1) intervention description, (2) evidence of success, (3) digital tool appropriateness and (4) implementation feasibility. Each thematic area had a set of questions that elicited open-ended, qualitative responses followed by questions that were quantitatively rated on a scale from 0 to 3, representing readiness for that domain, to assess the suitability of transfer and scale-up of a COVID-19 IIS for RI.

Based on the literature review and initial expert input, the team clarified the tool’s intended target users and intended use cases (Table 3).

**Table 3:** Target users and use cases for the CRIISTA tool

**Target users**	**Use cases**
Ministry of Health, Expanded Programme on Immunization, health management information system decision-makers and program managers	• Assess whether to transfer and scale up a COVID-19 IIS for RI • Guide planning for transfer and scale-up • Identify potential facilitators and barriers to successful transfer and scale-up
Implementing and technical partners	• Support collection and synthesis of information about the COVID-19 IIS and the RI context to present to decision-makers
Funders	• Assess whether and what to invest in the transfer and scale-up of a COVID-19 IIS for RI
Researchers	• Retrospectively assess suitability of a COVID-19 IIS for RI • Guide assessment of facilitators and barriers of successful transfer and scale-up

### Phase 2. Tool usability testing

#### Using CRIISTA (objective 1)

From August to December 2022, the implementation team completed the questions in the tool starting with a desk review, followed by a 1-hour expert panel interview.

A Ministry of Health report on Chanjo Kenya [41] served as the primary data source for the desk review, as few other documents were available regarding the system. From the desk review, the study team completed 13 of 43 (30%) of the questions in the tool. In November 2022, the implementation team facilitated a 1-hour expert panel interview via videoconference with four participants. Two were health informatics experts, one in a leadership role at the Kenya Ministry of Health’s Division of Health Informatics and another in a leadership role at a Kenyan software development and consultancy firm. Two others were immunization experts, one in a mid-level role at the Ministry of Health’s National Vaccines and Immunization Program Division and another at a large global health organization leading and supporting projects related to COVID-19 vaccination and IISs in Kenya. The interview began with an introduction to the project and the draft CRIISTA tool. Facilitators then collected data on 26 (60%) of the questions in the tool about Chanjo Kenya that were not answered via desk review. Four (9%) of the questions in the tool, intended for decision-makers to determine the suitability of the COVID-19 IIS for the RI context, were not answered by desk review or interview.

#### Evaluating CRIISTA’s usability and feasibility (objectives 2–3)

The implementation team identified that the process of answering questions in the CRIISTA tool through document review and interviews was appropriate and feasible. However, in Kenya, experts who could answer detailed questions about the COVID-19 IIS or RI context were not always the ones with the authority to decide to transfer Chanjo Kenya to the RI context, which is why the four questions mentioned previously were left unanswered. Thus, the implementation team recommended that once the information collection step is completed, that the information be synthesized and presented to key decision-makers during a facilitated consensus-building workshop.

The implementation team made several recommendations to improve the assessment tool’s usability, specifically related to its length, question order, question wording and the final assessment step. The team indicated that completing the full tool was time- and resource-intensive and recommended that, where possible, questions should be cut, shortened and changed from open-ended to close-ended. Though there were 43 total questions in the tool, many had open-ended sub-questions (e.g. ‘Describe the routine immunization data entry, management or use problem that the proposed intervention seeks to address, including the scope of the problem, e.g. Who is affected? How widespread is the problem? What is known about the causes?’), which often led to interview participants answering part, but not all, of the questions.

The team also found that ordering questions by scalability-based thematic area (i.e. intervention description, evidence of success, digital tool appropriateness and implementation feasibility) meant that discussions would skip around, for example, from technology to policies then back to technology, and that this was a barrier to the flow of the panel interview. Thus, the team recommended that the questions be organized and ordered by IIS-topic area, such as context, technology and users.

Finally, the team recommended that the tool end with a final score and interpretation for whether the IIS was suitable for transfer and scale-up to ensure the tool leads to a clear decision. Improving usability of the tool was also expected to improve feasibility of using the tool.

### Phase 3. Tool revision and user guide development

#### Tool structure and questions

Three key changes were made to the CRIISTA tool based on the usability testing to improve the tool’s usability (Table 4).

**Table 4:** Summary of key changes made to the CRIISTA tool after usability testing

Key change	Draft	Revised	Rationale
Thematic areas	Scalability-based: • Intervention description • Evidence of success • Digital tool appropriateness • Implementation feasibility	Immunization information system-based: • Context • Functionality • Technology • Users • Resources	**Increase usability and usefulness by…** • Tailoring the conversation specifically to IISs rather than all digital health interventions for RI data management • Improving flow of discussion • Facilitating in-depth discussion • Asking targeted questions to experts (e.g. technology questions to technology experts and policy questions to policy experts) • Enabling more direct comparisons between the COVID-19 IIS and the needs related to an IIS for routine immunization
Question format	Primarily a list of open-ended questions and sub-questions, e.g. ‘Describe the technological infrastructure requirements for the digital tool and whether they are adequate to support the tool’s transfer to routine immunization (e.g. availability of IT equipment, electricity, internet connectivity, data server capacity to support increased data flow, processing and storage, etc.)’	Primarily one closed-ended question at a time with response choices, e.g. ‘What hardware is required for the COVID-19 IIS? • Desktop/laptop • Tablet • Phone • Printers • Surge protectors • Wi-Fi routers • Solar panels • Other’	**Increase usability, feasibility and data quality by…** • Reducing time and effort required to complete the tool by providing response options • Increasing data completeness by only asking one question at a time
Tool output	Seven quantitative questions ranked on a 0–3 scale (not at all, to a small extent, somewhat, to a large extent and not applicable) to prompt discussion among decision-makers on the suitability of the COVID-19 IIS to transfer and/or scale-up for the RI context	Average thematic area domain score calculated based on 12 quantitative questions ranked on a 5-point Likert scale (strongly agree, agree, neutral, disagree, strongly disagree and not applicable) with guidelines on how to interpret the scores	**Increase usefulness by…** • Increasing decision clarity

First, the team decided to narrow the focus of the tool from any digital health interventions related to vaccine data management to electronic IISs. During useability testing and through other conversations with national-level decision-makers, we recognized that electronic immunization registries—a form of electronic IISs—were the primary digital health interventions being considered for scale-up. CRIISTA had an opportunity to inform these decisions, which had significant resource and impact considerations for countries; however, feedback suggested that a tool targeted to IISs would be more useful and impactful. With this in mind, we changed the thematic areas from four scalability-based themes (intervention description, evidence of success, digital tool appropriateness and implementation feasibility) to IIS-based themes (context, functionality, technology, users and resources) to better align with other readiness assessments of IISs and to improve the flow of the questions during discussion.

Second, individual questions in the tool were changed from multiple open-ended questions to single, closed-ended questions with response choices. While the number of questions in the tool appeared to nearly double from 43 in the draft tool, the questions were simplified to only ask one question at a time and minimize the time and effort required to complete them. The questions encouraged systematic reflection on the needs and requirements for an improved RI IIS so that any potential gaps in what exists, and what is needed, are immediately apparent. The narrowed focus on IISs allowed the questions to more directly determine whether the known technical and resource requirements of an RI IIS could be met. For example, most COVID-19 IISs do not exchange data with civil registration and vital statistics databases, but this is a functionality required to be able to monitor vaccination coverage of all children—an important goal of improved IISs. Important questions pertaining to resource needs remain in the tool, but the focus on IISs allows more specificity on the known hardware and human resource requirements for IISs, such as at least one tablet and staff with digital health literacy per vaccinating health facility. With the more recent movement towards integrating COVID-19 vaccination data into RI HISs as part of service delivery integration efforts, the revised tool explores these contextual factors to enable transparency across those conversations as well.

Lastly, the output of the tool was revised such that completion of the tool would result in a clear suitability assessment score for each theme. The revised version has a set of three to five high-level statements for each domain that help synthesize responses to assess the overall alignment between the COVID-19 IIS and the RI needs and context, and the suitability for transferring the COVID-19 IIS for RI. The CRIISTA tool, user guide, and corresponding workshop facilitation materials include guidelines on how to interpret the scores and identify recommendations for next steps (e.g. whether to scale up or transfer the COVID-19 IIS for RI).

#### Proposed process for implementing CRIISTA

Based on usability testing, the team revised the suggested three-step process for implementing the CRIISTA tool [42]. These steps are summarized as follows:

- **Step 1. Set-up and planning.** In this step, the implementation team develops a work plan; identifies key decision-makers, implementers and experts to engage and interview; and adapts the tool questions to meet the specific needs of the context, implementation team and stakeholders. An important part of this step is to identify the key people to engage in each step to ensure that technical experts are engaged for the information collection step and decision-makers are engaged for the suitability assessment step; in some contexts these may be the same people while in others they may differ, as was the case in Kenya.
- **Step 2. Information collection and synthesis.** In this step, the implementation team gathers and reviews available documents and conducts key informant interviews with the stakeholders identified in Step 1 to gather information about the COVID-19 IIS and the current and desired state of the IIS for RI. The implementation team reviews the information collected and completes the tool questions.
- **Step 3. Suitability assessment workshop.** In this last step, the implementation team summarizes the responses to questions and qualitative insights for each thematic area in a slide template provided in the user guide. The team facilitates a workshop discussion with decision-makers to review the summarized responses alongside the suitability assessment questions. The average suitability score for each thematic area is calculated through guided discussion and the scores are discussed together to inform a decision and recommendations for next steps.

## DISCUSSION

The COVID-19 pandemic accelerated the creation and adoption of new IISs to record, manage and monitor COVID-19 vaccination data, which presented opportunities to immunization decision-makers to consider how they might leverage these investments for RI. However, as implementation progressed, it became clear that many IISs faced implementation challenges and were not always built with interoperability and sustainability in mind [43]. This led national-level decision-makers to question whether and how these constrained systems and their functionality could be transferred to RI, which itself has faced issues in introducing and scaling up IISs [11–13]. We developed the CRIISTA tool and assessment process to support these decisions.

CRIISTA is unique among existing digital health tools given its focus on assessing the suitability of a digital health intervention in one context for use in another context. While there are a number of existing digital health assessment tools, nearly all tools focus on the digital health enabling environment rather than on a specific digital health intervention. These tools assess factors such as country readiness according to key digital health building blocks [10], which are relevant for understanding whether a country has the necessary enabling environment for digital health but lack the specificity needed to examine the implementation of a particular digital health intervention, or the specific case of transferring it from one funding and programmatic context to another. In this way, the CRIISTA tool fills a gap by providing decision-makers with a set of considerations to rapidly and systematically assess whether a COVID-19 IIS is suitable for meeting RI needs before investing extensively in the steps required to plan and implement transfer and scale-up.

While CRIISTA was uniquely designed to meet the specific needs expressed by national governments and their partners, it aligns with other digital health tools [21–24, 31–40] in that it includes assessment categories and questions that relate to the WHO and International Telecommunication Union national eHealth strategy building blocks [10]. The CRIISTA tool builds on the health system elements that are identified as vital for successful introduction and scale-up of digital interventions in existing digital health frameworks, and incorporates select questions from validated, widely used and accepted tools, increasing the potential validity and usefulness of the CRIISTA tool. Not only will this alignment allow for users of the tool to focus on a minimum set of considerations for a rapid but well-informed decision about transfer and scale-up, it also prepares users in planning for transfer and scale-up. For example, if users determine that a COVID-19 IIS is suitable to transfer for the RI context and meets RI functional requirements, the next step may be to plan for transfer using the electronic immunization registry practical considerations guide [18]. Much of the information needed for planning will already have been collected and synthesized through the use of the CRIISTA tool.

The concept and components of the CRIISTA tool align closely with the USAID COVID-19 Digital Health Collaborative Learning Agenda Theory of Change [17, Figure 1], particularly the hypothesis that the immediate outcomes that support the COVID-19 digital health response (e.g. data use, information systems and processes) can facilitate strengthening of the broader HIS ecosystem, including RI HISs. This paper highlights that for investments to contribute to longer-term outcomes, a careful analysis of the digital health enabling environment is necessary, along with a clear understanding of how that enabling environment and broader context differs between COVID-19 and the broader health system. While the theory of change assumes that COVID-19 investments will strengthen the digital health enabling environment, and thus longer-term systems strengthening, the review and experience of developing and implementing CRIISTA suggest that improvements to the enabling environment because of COVID-19 investments may not reach other health areas. System-wide digital maturity is likely an important predicator as to whether a COVID-19 digital investment will be institutionalized.

While the CRIISTA tool was originally developed to support assessment and decision-making of transferability of COVID-19 IISs for RI, it may have additional use cases for other digital health interventions or future health emergencies, given that the core element of the tool is to support rapid, systematic decision-making that considers the digital health enabling environment building blocks for sustainable success. For example, future multi-country analysis of applications of the CRIISTA tool could build greater knowledge of the characteristics of COVID-19 IISs and country context that facilitate and hinder transfer for RI. Additionally, the CRIISTA tool may be used to assess the appropriateness and feasibility of transferring an existing IIS (such as for RI) for future health threats (such as mpox). CRIISTA may also be used to inform planning of future IIS innovations, as the tool facilitates assessment of an IIS and its fit and sustainability within the context of the broader HIS ecosystem. Finally, CRIISTA could be adapted to inform scale-up questions for other COVID-19 digital health interventions beyond IISs. Future studies may assess the appropriateness of the CRIISTA tool for these additional use cases and suggest necessary adaptations to the tool.

### Limitations

The development of the CRIISTA tool had some limitations. While the tool was developed, tested and revised with extensive consultation with immunization, digital health and health system experts, the process focused on improving the usability of the tool and refining the implementation of the process without evaluating the construct validity of the tool questions. Relatedly, testing of the CRIISTA tool in Kenya did not result in a final decision regarding whether to transfer and scale Chanjo Kenya for the RI context, possibly due to the inconvenient timing of the CRIISTA implementation close to national elections, which limited the participation of key national-level decision-makers. As such, future studies should evaluate the construct validity of the CRIISTA questions as well as assess its effectiveness in identifying the key gaps that need to be addressed to support successful transfer and scale-up. At the time of writing this manuscript, less than 6 months following data collection in Kenya, the question of Chanjo Kenya’s transferability for RI remains on decision-makers’ agenda without a firm decision on whether to transfer it. One major concern is that the RI HIS infrastructure is not uniform across facilities in the country, and transfer of Chanjo Kenya for RI would risk running two parallel systems. That is, well-resourced facilities with electricity, internet and tablets could transition to Chanjo Kenya while facilities without these resources would continue to use a manual, paper-based system, further exacerbating resource equity issues. Effective implementation of the CRIISTA tool should prompt users to consider these types of infrastructure concerns and to bring key decision-makers together for a facilitated review and discussion of the data to inform decision-making.

While user feedback from this testing experience was helpful, it represents the feedback of a small set of stakeholders from one country. Further, the ever-evolving nature of the COVID-19 pandemic has meant that the needs of the health system and decision-makers are constantly changing. At the time of writing this manuscript, the priority related to COVID-19 vaccination was the need to integrate COVID-19 immunization services and data with RI or primary health care services and data systems. While the primary use case of the CRIISTA tool was to support decision-makers to assess whether to transfer and scale up a COVID-19 IIS for RI and not to support the planning of system integration, many of the questions in the CRIISTA tool may be useful for this newer need. To reflect this need for flexibility and adaptability, the user guide emphasizes the importance of making adaptations to the tool and implementation process during the first planning step and indicates opportunities for different approaches to the implementation process, depending on the tool’s use case.

## CONCLUSION

The CRIISTA tool meets a critical need and, by its design, seeks to contribute to increased country capacity to strengthen the eHealth building blocks and advance a country’s HIS architecture. The tool aims to contribute to improved vaccine equity by supporting countries to leverage their COVID-19 investments to build and scale up IISs that can contribute to identifying, monitoring and reaching zero-dose and under-immunized children, so that, ultimately, all children everywhere can benefit from life-saving vaccines.

## Data Availability

Data from the literature review is publicly available without restriction and included in this published article. Data from project documents and pause and reflect sessions are available upon request from the corresponding author. The final tool and user guide is publicly available at: https://usaidmomentum.org/resource/criista.
